# 

**Published:** 1890-04

**Authors:** 


					﻿io cts. a copy.
APRIL, 1890.
$i.do per-year.
HALLS
gj0VRN/\L‘J
HEALT Li
ESTABLISHED 1854
W 37
C^2r 4
OFFICE, 206 BROADWAY, EVENING POST BUILDING, Room 11, N. Y.
Entered as Second-Oass Matter at the Post-Office, New York.
				

